# Draft Genome Sequence of the Multidrug-Resistant Citrobacter freundii 132-2 Strain Isolated from a Domestic Duck in Bangladesh

**DOI:** 10.1128/mra.00378-23

**Published:** 2023-06-26

**Authors:** Tarana Ahmed, M. Saiful Islam, Mohammad Nuruzzaman, Mohammad Sadekuzzaman, S. M. Lutful Kabir, M. Tanvir Rahman, M. Shahidur Rahman Khan

**Affiliations:** a Department of Microbiology and Hygiene, Bangladesh Agricultural University, Mymensingh, Bangladesh; b Department of Livestock Services, Ministry of Fisheries & Livestock, Government of the Peoples Republic of Bangladesh, Dhaka, Bangladesh; c Ministry of Public Administration, Dhaka, Bangladesh; d Central Disease Investigation Laboratory (CDIL), Department of Livestock Services, Ministry of Fisheries & Livestock, Government of the Peoples Republic of Bangladesh, Dhaka, Bangladesh; DOE Joint Genome Institute

## Abstract

We sequenced a multidrug-resistant strain of Citrobacter freundii, 132-2, isolated from a cloacal swab sample of a domestic duck. The whole genome of the C. freundii 132-2 strain had a length of 5,097,592 bp, 62 contigs, two plasmids, and an average G+C content of 51.85%, with a 105.0× genome coverage.

## ANNOUNCEMENT

The extensive and inappropriate use of antimicrobial agents has resulted in the emergence of antimicrobial resistance in bacteria, leading to various multidrug-resistant (MDR) clones, which have become a major global public health concern (1, 2). Ducks have the potential to harbor antimicrobial-resistant and MDR pathogens that could transfer to humans due to their interactions with them (3). Citrobacter freundii is commonly found in the environment, soil, water, human clinical samples, and intestinal tracts of animals (4). C. freundii can be transmitted from ducks to humans through various means, such as contact with infected eggs, raw or undercooked meat, and the handling of duck carcasses at the slaughterhouse (3, 5). In humans, C. freundii isolates can cause bacteremia and severe neurological symptoms such as extreme cognitive impairment, seizures, and hemiparesis, and, in some cases, even death in children, so that it represents a considerable threat to human health (6, 7).

From January 2020 to January 2022, cloacal swab samples of domestic ducks (Anas platyrhynchos *domesticus*) were collected from the Kishoreganj district of Bangladesh and transported to our laboratory (24.7196°N, 90.4267°E). The specimens were then cultivated in nutrient broth (HiMedia, India), streaked on a xylose-lysine deoxycholate agar (HiMedia, India) plate, and incubated at 37°C for one night. Afterward, pure colonies were selected and subjected to staining and biochemical tests to isolate *Citrobacter* spp. (8). Finally, C. freundii was identified using a matrix-assisted laser desorption ionization–time of flight mass spectrometry assay (9). The MDR C. freundii isolate (resistant to >7 antimicrobial classes by disk diffusion method [10]) was incubated overnight in nutrient broth (HiMedia, India) at 37°C. The DNA was extracted from the collected culture using the Qiagen DNA minikit (Qiagen, Hilden, Germany). We used a NanoDrop 2000 UV-visible (UV-Vis) spectrophotometer (Thermo Fisher, Waltham, MA, USA) to determine the concentration of DNA present and its level of purity. To create the DNA libraries, the Nextera DNA flex library prep kit (Illumina, San Diego, CA, USA) was employed. The Illumina NextSeq2000 platform was used for whole-genome sequencing (WGS) (read length, 2 by 150 bp). The genome was assembled using Unicycler v0.4.9 (11). Before that, the raw paired-end reads (*n* = 7,855,310) were subjected to trimming using Trimmomatic v0.39 (12) and underwent quality assessment with FastQC v0.11.7 (https://www.bioinformatics.babraham.ac.uk/projects/fastqc/). The annotation of the genome was performed with Prokka v1.14.6 (13) and PATRIC (14). The PathogenFinder 1.1 (15), MLST 2.0 (16), PlasmidFinder-2.0 (17), CARD (18), VFDB (19), and RAST.v.2 (20) databases were utilized to identify the pathogenicity index, sequence type, plasmids, antimicrobial resistance genes (ARGs), virulence factor genes (VFGs), and metabolic functional features, respectively, in our assembled genome. All tools were run with default parameters.

The genome coverage of Citrobacter freundii 132-2 was 105.0×, and a total of 62 contigs were obtained. Our assembled genome had a total length of 5,097,592 bp, a GC content of 51.85%, and an *N*_50_ value of 536,796 bp. This assembled genome identified two important plasmid replicons, i.e., IncFIB(pHCM2) (110,089 bp with a 98.51% identity to AL513384) and IncR (16,190 bp with a 100% identity to DQ449578). Moreover, this genome consists of a sequence type of ST18 (using MLST 2.0) and a probability of being a pathogen for humans of 89.6% (using PathogenFinder 1.1). The strain carried 45 predicted ARGs under 19 antimicrobial categories and 83 predicted virulence genes under more than 12 predicted virulence factors. Moreover, Citrobacter freundii 132-2 contained 395 subsystems (having 2,298 genes) with 33% coverage, 5,024 protein-coding sequences, and 81 RNA genes.

The Animal Welfare and Experimentation Ethical Committee, which is the institutional ethics committee of Bangladesh Agricultural University in Mymensingh, granted approval for the procedures and protocols associated with this research under the reference number of AWEEC/BAU/2020(10).

### Data availability.

The whole-genome sequencing (WGS) shotgun study for Citrobacter freundii 132-2 was submitted to NCBI/GenBank with accession number JAPQWA000000000, and the raw reads were deposited under SRA accession number SRR24848516 (BioProject accession number PRJNA907483). The current version referred to this paper is identified as JAPQWA010000000.
